# Predictors of Curve of Spee Leveling in Class II Division 1 Malocclusion Treated With Reverse Curve of Spee Archwires: An Observational Study

**DOI:** 10.7759/cureus.67163

**Published:** 2024-08-19

**Authors:** Allu Harini, Aparna Kadiveti, Ganugapanta Vivek Reddy, Sai Sushma K Alahari, Gowri Sankar Singaraju, Prasad Mandava

**Affiliations:** 1 Orthodontics and Dentofacial Orthopedics, Narayana Dental College & Hospital, Nellore, IND; 2 Orthodontics and Dentofacial Orthopedics, St. Joseph Dental College, Eluru, IND

**Keywords:** molar, incisor, rcs wires, intrusion, extrusion, curve of spee

## Abstract

Introduction

Aligning and leveling is the initial stage of comprehensive fixed orthodontic treatment aimed at minimizing the depth of the curve of Spee (COS). Various techniques exist to decrease the magnitude of the curve. This study investigates the skeletal and dental factors that reduce COS in individuals with minor class II malocclusions receiving nitinol (NiTi) wires with a reverse curve of Spee (RCS).

Materials and methods

The data for this observational study was collected from a sample of 84 patients who had class II molar relations and were sequentially treated with RCS NiTi wires throughout the initial leveling and aligning phase. All patients with class II molar relationships underwent non-extraction procedures during the leveling phase. The COS was determined using digitalized dental models. Skeletal and dental characteristics that could impact COS were identified and quantified using digital lateral cephalograms and orthopantomograms recorded during the pre-treatment (T1) and post-leveling (T2) stages. After calibrating the radiographs and models, we acquired angular and linear data. The data was categorized based on gender, growth pattern, and initial alignment of the teeth. We analyzed the differences between the groups using an independent t-test and an ANOVA. A paired t-test was used to compare the difference in the dimensional values between (T1) and (T2) points. Following the correlation coefficient tests, the study used stepwise multiple linear regression analysis to assess the predictive value of independent factors on the COS. The results were considered statistically significant at p < 0.05.

Results

The COS decreased by -1.43 ± 0.68 mm, which is statistically significant (<0.001*). There is no significant difference in COS reduction between the categorical variables. Despite statistically significant differences in the parameters between pre- and post-treatment, the linear correlation between most of the variables and COS reduction ranged from very weak (<0.20) to weak (0.20-0.39).

Conclusions

The vertical extrusion of lower premolars and molars combined with the intrusion of lower incisors contributed to the reduction of the COS by RCS wires. There is a change in the orientation of the occlusal plane with the flattening of the COS.

## Introduction

The purpose of orthodontic treatment planning is to achieve desirable changes in the appearance and structure of the maxillary and mandibular bones, teeth, and facial tissues. This involves establishing the best possible alignment and movement of the teeth when the jaw is at rest and in motion [1,2]. Exploration by Andrews identified six key traits observed in non-orthodontic models with normal occlusion, termed “the six keys to normal occlusion” [3]. These traits gauge ideal static occlusal relationships, guiding contemporary orthodontic practices, of which the sixth key or curve of Spee (COS) is important for inter-arch occlusal considerations. The occlusal alignment of teeth establishes the curvature of the occlusal plane (OP), known as the COS, which begins with the cusp of the canine, follows the buccal cusp tips of the premolar and molar teeth, continues through the anterior border of the mandibular ramus, and ends at the anterior aspect of the mandibular condyle [4]. It plays a crucial role in masticatory efficiency and occlusal harmony. This curvature varies from flat to mild in non-orthodontic models. However, a natural tendency for the COS to deepen over time poses challenges, such as deep overbites and crowded lower anterior teeth [4].

An excessive COS is associated with deep bite malocclusions, particularly in the case of class II malocclusions. The curve must exhibit a relatively mild configuration to establish a stable occlusion with appropriate excursive movements. Orthodontic treatment aims to address variations in the COS, with leveling techniques commonly employed to establish a flat or slightly reversed occlusal curvature, facilitating optimal interdigitation and canine relationships. This approach ensures occlusion stability post-treatment [5]. Clinicians should strive for a COS that does not exceed a depth of 1.5 mm to reduce occlusal interferences and promote ease of intercuspation. The treatment of deep-bite might involve intrusion of maxillary anterior teeth, intrusion of mandibular anterior teeth, extrusion of maxillary and mandibular posterior teeth, or any such combination [6]. Schudy advocated that the deep bite and deep COS be corrected by the extrusion of molars because the intrusion of anterior teeth has a high potential for relapse [7]. In his assessment of the post-treatment stability of deep-bite malocclusion, Cordon found that the extrusion of molars was stable in both maxillary and mandibular arches [8]. One commonly employed method for leveling this curve is the reverse curve of Spee (RCS) nitinol (NiTi) wires during the initial stages of treatment. The literature regarding the effects of RCS wires during leveling is scarce.

The current study aims to unravel the nuanced alterations in the mandibular arch parameters attributed to the correction of the COS in mild class II malocclusions. The research endeavors to comprehensively understand the factors contributing to the treatment changes. The study hypothesizes no correlation between COS leveling and incisor inclination, condylar guidance (CG), and arch width changes, as well as no difference in the effect of COS leveling across different vertical growth patterns when treated without extractions. By elucidating the relationships between COS leveling and various orthodontic parameters, this study aims to contribute to the refinement of treatment strategies and enhance the predictability of treatment outcomes.

## Materials and methods

The Institutional Ethical Committee of Narayana Dental College & Hospital, Nellore, India, reviewed and approved this observational analytical study (approval number ICE/NDCH/2022/MAR/P-24). An a priori computed sample size of at least 70 participants was estimated to assess pre- and post-treatment changes using multiple regression with a fixed model and R² deviation from zero analysis for 10 predictors. This calculation accounted for a 5% Type I alpha error and a 20% Type II beta error. The sample size was determined using G*Power version 3.1.9.6, with a standard deviation derived from a previous study, resulting in an effect size of 0.5 [9].

The data for this investigation was obtained using a combination of prospective data collection and retrieving stored retrospective records. The study exclusively included patients in the actively non-growing phase aged 18-25 years with COS measuring 2 mm or more. Patients exhibiting complete dentition except the third molars, a Class II molar relationship, and vertical incisor overlap were chosen for the study. All the patients had class I skeletal patterns with an ANB angle of 2-4 degrees. The study covered cases of anterior crowding, or spacing that did not exceed 3 mm. All patients underwent comprehensive orthodontic treatment using fixed appliances, including non-extraction cases and extraction cases that required alignment before the removal of premolars for orthodontic purposes. The patients not included in the study had an open bite, retroclined upper incisors, anterior and posterior crossbites, periodontal diseases, tooth abnormalities, and improper dental restorations. Exclusions were made for asymmetries, temporomandibular joint problems, and craniofacial abnormalities.

The patients received treatment utilizing stainless steel (SS) metal brackets with an MBT prescription of 0.022 × 0.028-inch slot size (Mini Diamond™, Ormco, Brea, CA, USA). The COS was leveled in all patients using typical NiTi archwires (G4™ Nickel Titanium, G&H Orthodontics, G&H Wire Company, Franklin, IN, USA) in a standardized sequence of 0.014, 0.016, 0.018-inch, and 0.017 × 0.025-inch. Subsequently, 0.017 × 0.025-inch, 0.019 × 0.025-inch NiTi, and 0.019×X 0.025-inch RCS wires were used until a 0.019 × 0.025-inch SS wire could be inserted passively for retraction. The treatment models, lateral cephalogram, and orthopantomogram (OPG) were analyzed at the initiation of treatment T1 (pre-treatment) and after the leveling and aligning process just before the SS wires were inserted (T2).

Digital lateral cephalograms of the subjects were obtained using an operating machine fixed with a cephalostat (Model No. MR05, type 84086511, 2003, Villa Sistemi Medicali, Italy). The OPG was taken with the digital OPG (Promax, Model 2008, Planmeca, Helsinki, Finland). All the radiographs were taken with the same machine, with an inherent magnification error of 1.13 for lateral cephalograms and 1.12 for OPG. The soft copies of the OPG and lateral cephalograms were transferred from their proprietary formats and saved as readable image files (JPEG) to the computer desktop software system (Dolphin Imaging premium version 11.95, April 2019®, Chatsworth, CA, USA) for calibration and recording the measurements. The three-dimensional (3D) images of the conventional models were scanned and captured by the 3D scanner in stereolithographic format (3D Auto Scan-DS-EX Dental 3D Scanner, Shining 3D, Hangzhou, China).

The digitalized soft copy images of the lateral cephalogram, OPG, and the models were calibrated using the two-step grid method utilizing graph paper. The mesiodistal diameter of the first molar on the physical model was used as a reference for calibration. The standard grid of graph paper with each square of 1 mm was superimposed with 1:1 magnification on the precalibrated digital images, and the measurements could be read out directly on the screen and cross-checked with the grid measurements. The Dolphin Imaging premium version 11.95 was utilized for calibration and recording the measurements.

**Figure 1:**
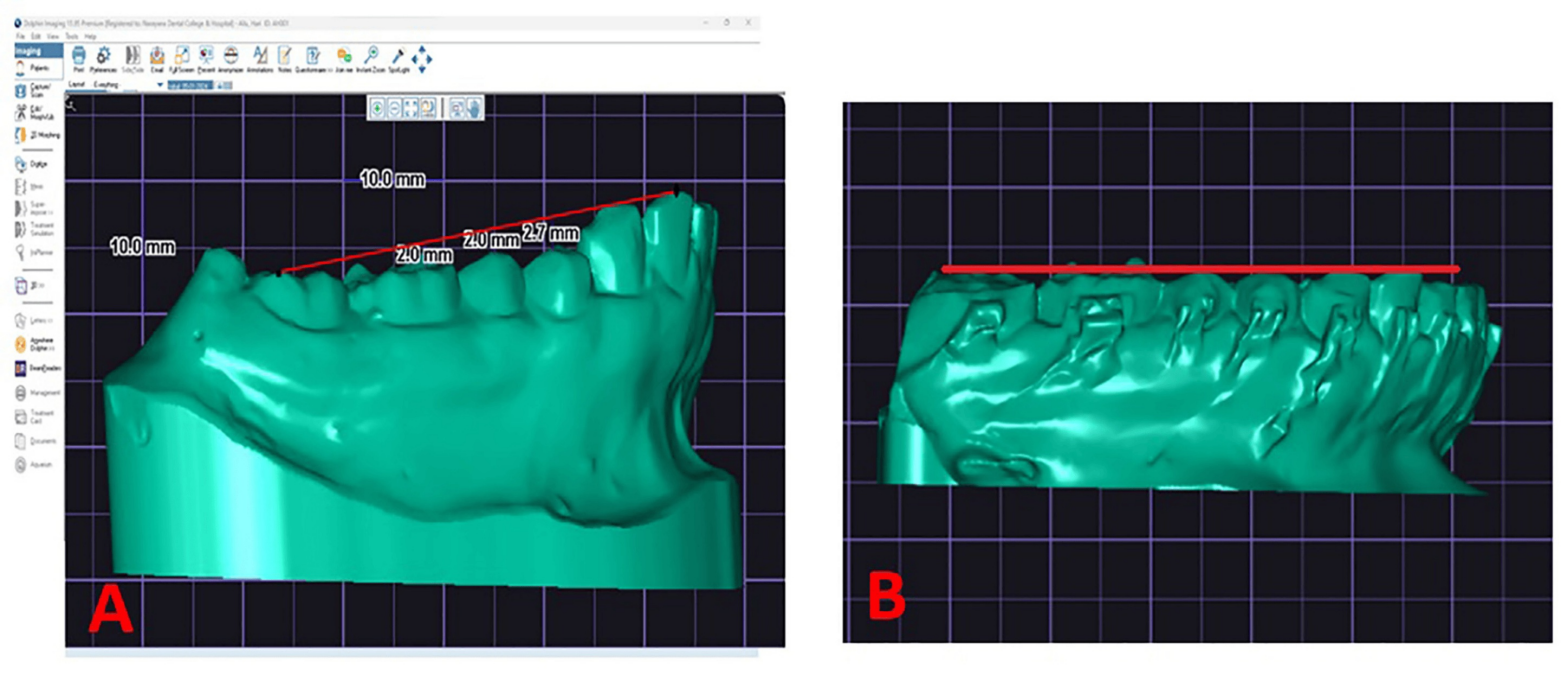
Pre-treatment and post-treatment measurement of COS on digital models in Dolphin Imaging premium version 11.95 COS: curve of Spee

**Figure 2:**
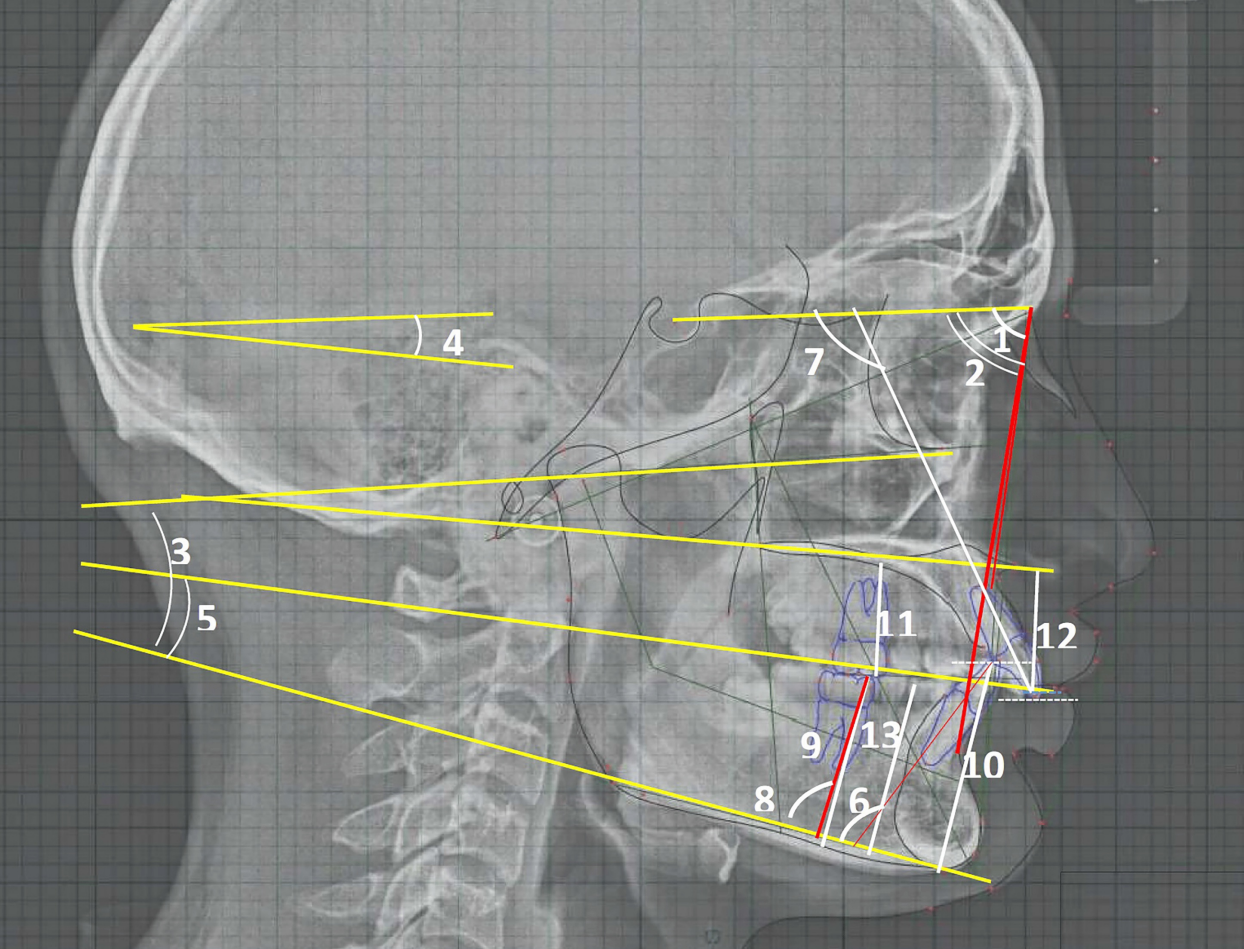
Skeletal and dental – angular and linear measurements on digitized lateral cephalogram 1. SNA: angle sella point A (angle); 2. SNB: angle sella point B (angle); 3. FMA: Frankfurt horizontal-mandibular plane angle; 4. SN-OP: sella nasion plane-occlusal plane angle; 5. OP-MP: occlusal plane-mandibular plane angle; 6. L1-MP: lower incisal inclination to mandibular plane (angle); 7. U1-SN: upper incisor-mandibular plane angle; 8. L6-MP: lower first molar-mandibular plane angle; 9. L6-MP (mm): lower first molar-mandibular plane distance; 10. L1-MP (mm): lower incisor-mandibular plane distance; 11. U6-PP (mm): upper first molar-palatal plane distance; 12. U1-PP (mm): upper incisor-palatal plane distance; 13. PM-MP (mm): premolar(first)-mandibular plane distance

**Figure 3:**
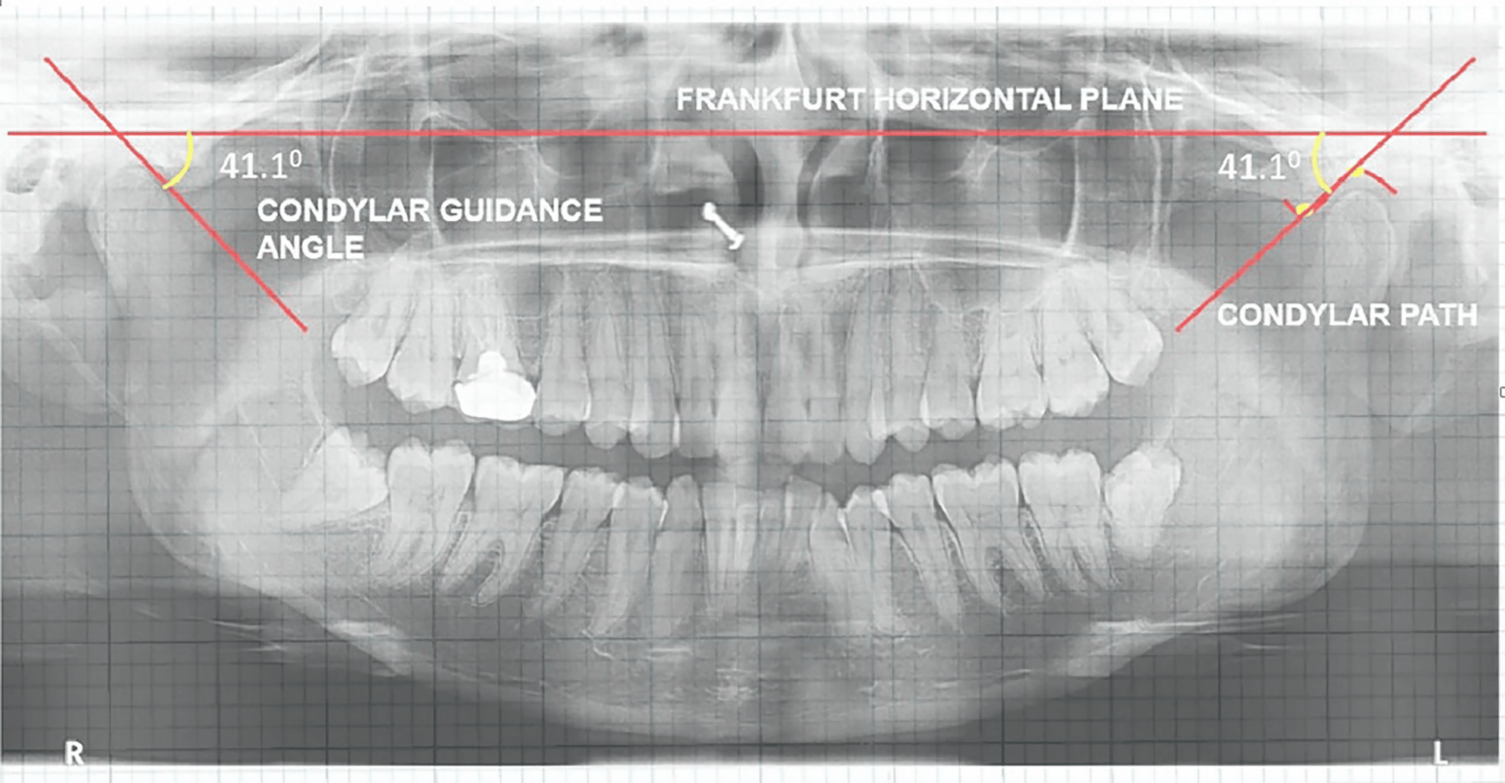
Measurement of CG angle on digitized OPG CG, condylar guidance; OPG, orthopantomogram

**Figure 4:**
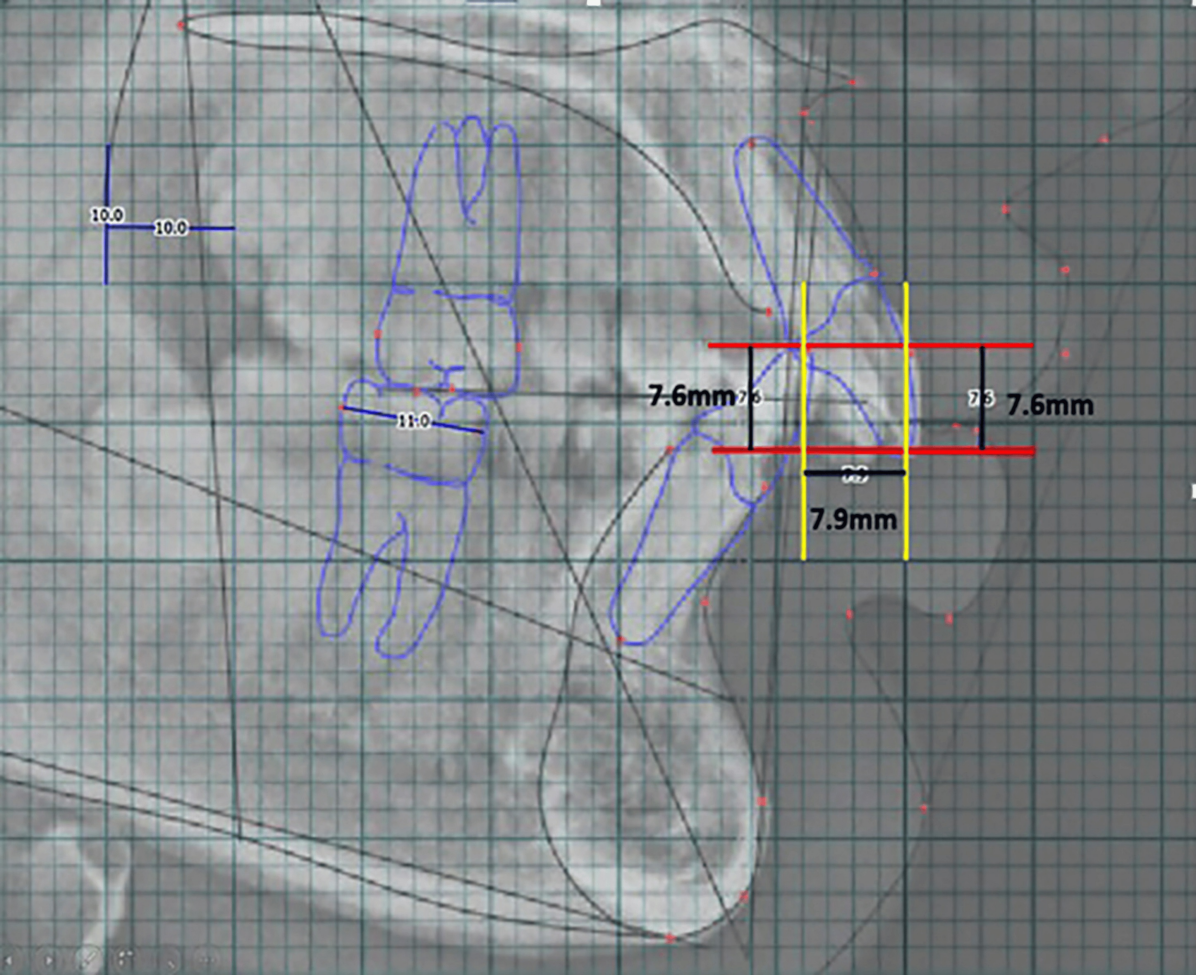
Horizontal and vertical overlapping between upper and lower incisors – overjet and overbite in mm

**Figure 5:**
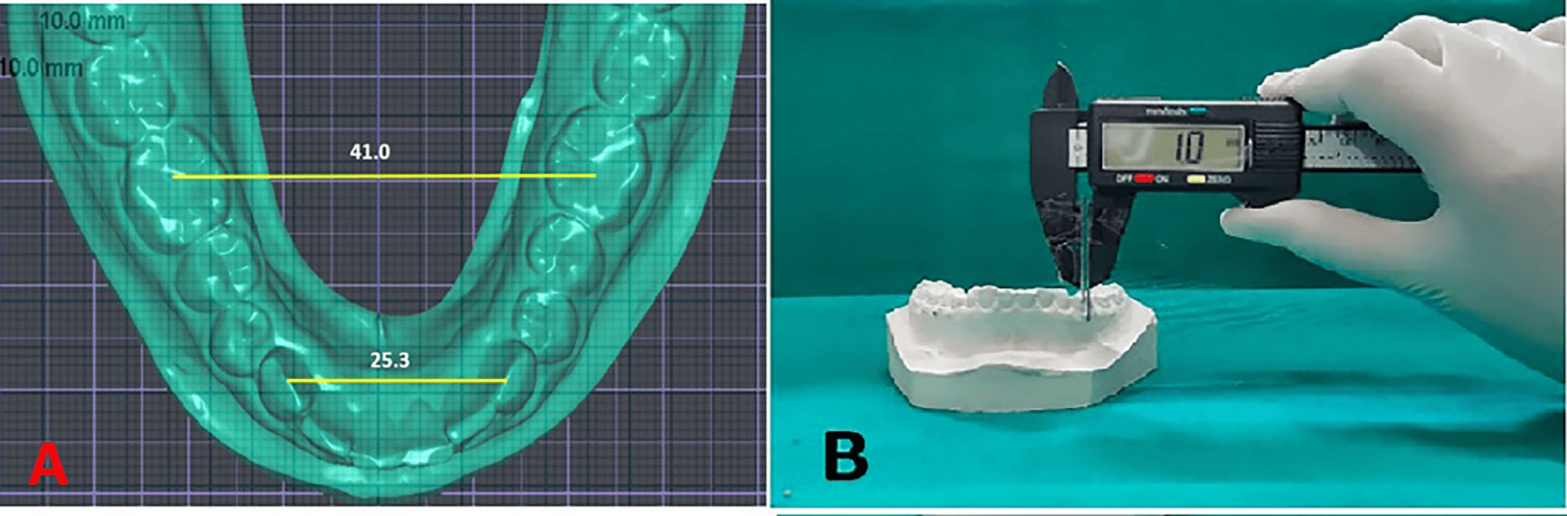
(A) Transverse dimensions – inter-canine width and inter-molar width on the lower arch. (B) WALA-FA on the lower first premolar and molar FA: facial axis; WALA: Willy Andrews Larry Andrews

Table 1 depicts the parameters measured in the study. The COS, intercanine width (ICW), and intermolar width (IMW) were measured using the digital study model. The WALA-FA premolar and the WALA-FA molar measurements were made directly on the physical study models. The average of the left and right sides was taken for these parameters. The CG was measured on the OPG as described in the earlier study [10]. All the remaining dental and skeletal parameters were obtained from the lateral cephalograms (Figure 1, Figure 2, Figure 3, Figure 4, Figure 5). The inter-examiner and intra-examiner reliability of two examiners (AH and GV) demonstrated a mean percentage agreement of 95% and 99% for linear and 93% and 95% for angular measurements. Dahlberg’s formula [11] test showed reproducibility of 99% for skeletal parameters and 95% for the measured dental parameters on the lateral cephalogram.

**Table 1:** Parameters measured in the study CG, condylar guidance; COS: curve of Spee; FA: facial axis; FH: Frankfurt; FHP: Frankfurt horizontal plane; FMA: Frankfurt horizontal mandibular plane angle; ICW: intercanine width; IMW: intermolar width; MP: mandibular plane; OP: occlusal plane; PP: palatal plane; SN: sella-nasion; SNA: angle sella point A; SNB: angle sella point B; SN-OP: sella-nasion plane-occlusal plane angle; WALA-PM: Willy Andrews Larry Andrews point lower first premolar

S. no	Parameter measured	Units of measurement	Description
1	Depth of COS	Linear/mm	The distance from the deepest cusp tip to the OP
Skeletal parameters – sagittal			
2	SNA	Angular/degrees (^o^)	The angle between the SN plane and point A
3	SNB	Angular/degrees (^o^)	The angle between the SN plane and point B.
4	ANB	Angular/degrees (^o^)	The difference between angle SNA and angle SNB
5	FMA	Angular/degrees (^o^)	The angle between the FH plane and the mandibular plane.
6	SN-OP	Angular/degrees (^o^)	The angle between the SN plane and the OP
7	OP-MP	Angular/degrees (^o^)	The angle between the OP and MP
Skeletal parameters – vertical			
8	CG	Angular/degrees (^o^)	The angle between the FHP and the constructed condylar path
Dental parameters			
9	L6-MP	Angular/degrees (^o^)	The angle between the long axis line mandibular first molar (L6) and MP
10	U1-SN	Angular/degrees (^o^)	The angle between the SN plane and the long axis of the upper central incisor (U1)
11	L1-MP	Angular/degrees (^o^)	The angle between the long axis of the lower central incisor (L1) and the MP
12	L6-MP	Linear/mm	Linear measurement of a perpendicular dropped from the mesiobuccal cusp of the lower first molar (L6) to the MP
13	L1-MP	Linear/mm	Linear measurement of a perpendicular dropped from the crown tip of the lower incisor (L1) to the MP
14	U6-PP	Linear/mm	Linear measurement of a perpendicular dropped from the mesiobuccal cusp tip of the upper first molar (U6) PP
15	U1-PP	Linear/mm	Linear measurement of a perpendicular dropped from the crown tip of the upper incisor (U1) to the PP
16	PM-MP (lower first premolar-mandibular plane)	Linear/mm	Linear measurement between the crown tip of the lower first premolar and the mandibular plane
17	ICW	Linear/mm	Distance between the tips of the cusps of the right and left mandibular permanent canines
18	IMW	Linear/mm	Distance between the central fossa of the right and left mandibular permanent first molars
19	WALA-FA premolar	Linear/mm	The horizontal distance between the WALA ridge and the FA point of the mandibular first premolar
20	WALA-FA molar	Linear/mm	The horizontal distance between the WALA ridge and the FA point of the mandibular first molar
21	Overjet	Linear/mm	Horizontal overlap between the upper and lower incisors
22	Overbite	Linear/mm	Vertical overlap between the upper and lower incisors

Statistical analysis

All the collected data was entered into a Microsoft Excel sheet. Continuous variables are presented as mean and standard deviation as they follow a normal distribution, as confirmed by the Kolmogorov-Smirnov test. The correlation between the dependent continuous variable COS and other independent continuous variables was assessed using Pearson’s coefficient of correlation test. The relationship between categorical variables and the COS was evaluated using the biserial correlation and the Spearman correlation. A three-way ANOVA was conducted to examine the interactive effect of categorical variables on the COS. Multiple linear regression was performed to identify predictive variables for the dependent variable of COS. Before running the regression model, the variance inflation factor (VIF) was used to check for multicollinearity between continuous variables. All statistical analyses were done using statistical software (DATAtab online statistics calculator, DATAtab e.U. Graz, Austria). The statistical significance level for all analytical tests was set at p-values <0.05.

## Results

The final run analyzed 84 cases in total. It included 57 females and 27 males. The average age of the patients is 22.3 ± 1.2 years, and the average time of leveling ranges from 2 to 5.5 months. The data collected included 22 continuous variables. The variable FMA was further ranked in order as a categorical variable, with FMA angles below 20° considered as horizontal growth patterns, 20-25° as average growth patterns, and above 25° considered as vertical growers. The data was further categorized as crowding and spacing for analytical purposes based on the initial alignment. The categorical-wise distribution of the COS shows no significant differences (Table 2). A comparison of the parameters between class II pre-treatment (T1) and post-treatment (T2) values shows a statistically significant COS decrease from 2.97 ± 0.79 mm to 1.54 ± 0.47 mm post-treatment. Other parameters SN-OP (o), OP-MP (o), L6-MP (o), L6-MP (mm), PM-MP (mm), ICW (mm), and IWM (mm), exhibited statistical significance (Table 3). The correlation between the variables that have cause and effect on COS is displayed in Table 4. Despite statistically significant differences in the parameters between pre- and post-treatment, the linear correlation between most of the variables and COS reduction ranged from very weak (<0.20) to weak (0.20-0.39). The strength of association is moderate for LI-MP (o), WALA-M, OJ, and OB. The association of the WALA-M (mm), OJ (mm), and OB (mm) with the flattening of the COS from pretreatment to post-treatment is positive, and the correlation is statistically significant. Other variables ANB, FMA, SN-OP, OP-MP, CG, L1-MP (mm), PM-MP (mm), ICW (mm), IMW (mm), and WALA-PM (mm) exhibited a positive correlation, but that is not statistically significant.

**Table 2:** COS category-wise comparison of pre-treatment and treatment changes * ANOVA for growth pattern; independent Student’s t-test for alignment and gender COS, curve of Spee

Category	n	Pre-treatment (T1)	Post-treatment (T2)	Treatment effects (T2-T1)	Pre-treatment comparisons (T1)	Treatment effects comparisons (T2-T1)
		Mean ± SD	Mean ± SD	Mean ± SD	Test static*	Test static*
Gender						
Female (reference)	57	2.89 ± 0.8	1.45 ± 0.46	-1.44 ± 0.68	T = -0.78; p = 0.44	t = -0.12; p = 0.22
Male)	27	3.14 ± 0.79	1.72 ± 0.49	-1.42 ± 0.72		
Growth pattern						
Horizontal (reference)	15	2.87 ± 0.62	1.42 ± 0.45	-1.51 ± 0.79	F = 2.67; p = 0.89	F = 0.66; p = 0.53
Average	27	3.44 ± 0.96	1.81 ± 0.59	-1.63 ± 0.81		
Vertical	42	2.71 ± 0.62	1.41 ± 0.34	-1.3 ± 0.54		
Alignment						
Crowding (reference)	63	3 ± 0.85	1.48 ± 0.46	-1.53 ± 0.72	T = -03.69; p = 0.75	F = 0.06; p = 0.91
Spacing	21	2.89 ± 0.64	1.73 ± 0.5	-1.16 ± 0.47		

**Table 3:** Comparison between class II pre-treatment and post-treatment parameters Test statistic: paired t-test; *p-values less than 0.05 are statistically significant -Ve values indicate a decrease in values, and +Ve values indicate an increase in values from pre-treatment to post-treatment. ANB (°): difference between SNA and SNB; CG: condylar guidance in degrees; COS: curve of Spee in mm; FMA (°): Frankfurt horizontal-mandibular plane angle; ICW (mm): intercanine width in lower arch; IMW (mm): intermolar width in lower arch; L1-MP (mm): lower incisor-mandibular plane distance; L1-MP (°): lower incisal inclination to mandibular plane in degrees; L6-MP (mm): lower first molar-mandibular plane distance; L6-MP (°): lower first molar-mandibular plane angle; OB: overbite in mm; OJ: overjet in mm; OP-MP (°): occlusal plane-mandibular plane angle; PM-MP (mm): premolar (first)-mandibular plane distance; SNA (°): angle sella point A in degrees; SNB (°): angle sella point B in degrees; SN-OP (°): sella-nasion plane-occlusal plane angle; U1-PP (mm): upper incisor-palatal plane distance; U1-SN (°): upper incisor-mandibular plane angle; U6-PP (mm): upper first molar-palatal plane distance; WALA-M: William Andrews Larry Andrews point lower first molar; WALA-PM: Willy Andrews Larry Andrews point lower first premolar

Sl. no	Variables	Parameter	Pre-treatment (T1) (n = 84)	Post-treatment (T2) (n = 84)	Treatment changes mean difference (T2-T1) (n = 84)	p
			Mean ± SD	Mean ± SD	Mean ± SD	
1	Dependent variable	COS (mm)	2.97 ± 0.79	1.54 ± 0.47	-1.43 ± 0.68	<0.001*
2	Independent variables – skeletal	SNA (^0^)	83.78 ± 3.85	83.7 ± 3.38	-0.08 ± 1.73	0.81
3		SNB (^0^)	79.01 ± 3.56	79.02 ± 3.11	0.01 ± 1.37	0.97
4		ANB (^0^)	4.76 ± 2.09	4.68 ± 1.72	-0.09 ± 1.36	0.74
5		FMA (^0^)	26.18 ± 6.99	26.68 ± 6.22	0.5 ± 2.29	0.25
6		SN-OP (^0^)	16.4 ± 5.57	13.35 ± 5.36	-3.05 ± 4.06	<0.001*
7		OP-MP (^0^)	16.06 ± 3.66	17.89 ± 3.96	1.82 ± 4.59	0.04*
8		CG (^0^)	43.74 ± 6.25	45.18 ± 5.2	1.44 ± 4.94	0.13
9	Independent variables – dental	LI-MP (^0^)	103.25 ± 9.1	105.73 ± 6.83	2.48 ± 6.87	0.05*
10		U1-SN (^0^)	111.4 ± 9.24	107.93 ± 7.17	-3.46 ± 9.53	0.06
11		L6-MP (^0^)	98.28 ± 5.39	100.42 ± 5.08	2.14 ± 5.64	0.05*
12		L6-MP (mm)	26.39 ± 2.74	27.17 ± 2.21	0.78 ± 2.03	0.05*
13		L1-MP (mm)	35.92 ± 3.27	35.17 ± 2.76	-0.75 ± 2.23	0.08
14		U6-PP (mm)	19.69 ± 2.24	19.8 ± 1.99	0.11 ± 1.72	0.74
15		U1-PP (mm)	25.45 ± 2.44	26.42 ± 2.28	0.98 ± 2.19	0.02*
16		PM-MP (mm)	23.58 ± 2.64	24.52 ± 2.52	0.94 ± 1.3	0.001*
17		ICW (mm)	25.41 ± 2.66	27 ± 2.09	2.5 ± 5.88	0.017*
18		IMW (mm)	41.7 ± 2.85	43.14 ± 2.26	2.93 ± 7.79	<0.001*
19		WALA-M (mm)	1.78 ± 0.54	1.28 ± 0.4	-0.5 ± 0.61	0.18
20		WALA-PM (mm)	0.51 ± 0.43	0.62 ± 0.36	0.11 ± 0.58	0.82
21		OJ (mm)	4.14 ± 2.51	2.48 ± 1	-1.67 ± 2.07	0.8
22		OB (mm)	4.05 ± 1.87	2.54 ± 0.95	-1.51 ± 1.35	1.12

**Table 4:** Coefficient of correlation between changes in the COS and other variables under study * p-values less than 0.05 are statistically significant; r: Pearson correlation coefficient for continuous variables; rpb: point-biserial correlation coefficient for binary variables; rs: Spearman correlation for ordinal variables ANB (°): difference between SNA and SNB; CG: condylar guidance in degrees; COS: curve of Spee in mm; FMA (°): Frankfurt horizontal-mandibular plane angle; ICW (mm): intercanine width in lower arch; IMW (mm): intermolar width in lower arch; L1-MP (mm): lower incisor-mandibular plane distance; L1-MP (°): lower incisal inclination to mandibular plane in degrees; L6-MP (mm): lower first molar-mandibular plane distance; L6-MP (°): lower first molar-mandibular plane angle; OB: overbite in mm; OJ: overjet in mm; OP-MP (°): occlusal plane-mandibular plane angle; PM-MP (mm): premolar (first)-mandibular plane distance; SNA (°): angle sella point A in degrees; SNB (°): angle sella point B in degrees; SN-OP (°): sella-nasion plane-occlusal plane angle; U1-PP (mm): upper incisor-palatal plane distance; U1-SN (°): upper incisor-mandibular plane angle; U6-PP (mm): upper first molar-palatal plane distance; WALA-M: William Andrews Larry Andrews point lower first molar; WALA-PM: Willy Andrews Larry Andrews point lower first premolar

Variable		Correlation coefficient	p-value significance (two-tailed)
Dependent variable	COS	1	
Categorical variables	Gender ^rpb^ (binominal variable) (reference: female/male)	0.01	0.95
	Growth pattern ^rs^ (ordinal variable) (reference: horizontal/average/vertical)	-0.12	0.53
	Alignment ^rpb^ (binominal variable) (reference: crowding/spacing)	0.24	0.21
Continuous variables ^r^	SNA (^o^)	-0.05	0.8
	SNB (^o^)	-0.22	0.25
	FMA (^o^)	0.22	0.26
	SN-OP (^o^)	0.07	0.7
	Op-MP (^o^)	0.23	0.24
	CG (^o^)	0.05	0.8
	LI-MP (^o^)	-0.36	0.06
	UI-SN (^o^)	-0.08	0.67
	L6-MP (^o^)	0.07	0.73
	L6-MP (mm)	-0.27	0.15
	LI-MP (mm)	0.02	0.92
	U6-pal (mm)	-0.2	0.33
	UI-pp (mm)	-0.07	0.73
	PM-MP (mm)	0.12	0.53
	ICW (mm)	0.01	0.96
	IMW (mm)	0.1	0.6
	WALA-M (mm)	0.4	0.03*
	WALA-PM (mm)	0.17	0.38
	OJ (mm)	0.39	0.038*
	OB (mm)	0.57	0.002*

A multiple linear regression analysis was performed to examine the influence of the categorical variables and all the continuous variables whose absolute coefficient values of correlation are above 0.2 or those that exhibit statistical significance (Table 5). The categorical variables were dummy-coded before entering the regression model. We conducted a multicollinearity assessment to evaluate potential issues within the predictive model. The results indicated tolerance values above the critical threshold of 0.10 and VIF values below 10, suggesting that multicollinearity is not a significant concern. These findings suggest that the predictors in the model are not highly correlated, supporting the reliability of the regression analysis. The regression model showed that the included variables explained 61.72% of the variance from the COS (IIA) variable. We used an ANOVA to test whether this value differed significantly from zero. Using the present sample, it was found that the effect was not significantly different from zero (F = 1.74, p = 0.108, R2 = 0.62).

**Table 5:** Multiple regression coefficient model of the variables – cause and effect of flattening of COS * p-values less than 0.05 are statistically significant COS: curve of Spee in mm; FMA (o): Frankfurt horizontal: mandibular plane angle; L1-MP (o): lower incisal inclination to mandibular plane in degrees; L6-MP (mm): lower first molar: mandibular plane distance; OB: overbite in mm; OJ: overjet in mm; OP-MP (o): occlusal plane: mandibular plane angle; SNB (o): angle sella: point B in degrees; T1: class II pre-treatment; T2: class II post-treatment; T2-T1: difference between pre- and post-treatment values; WALA-M: William Andrews Larry Andrews point lower first molar

Parameters (T2-T1)	Unstandardized coefficients	Standardized coefficients	Standard error	t	p	95% CI for B	
Model	B	Beta				Lower bound	Upper bound
(Constant)	2.75		0.55	5.03	<0.001>	1.58	3.93
Alignment - spacing (reference: crowding)	0.1	0.06	0.35	0.3	0.77	-0.65	0.85
Growth pattern - average (reference: horizontal)	-0.04	-0.02	0.44	-0.09	0.92	-0.97	0.89
Growth pattern - vertical (reference: horizontal)	-0.66	-0.42	0.5	-1.31	0.21	-1.73	0.42
Gender - male (reference: female)	0.16	0.09	0.34	0.47	0.64	-0.56	0.88
SNB (^o^)	0.02	0.03	0.13	0.14	0.89	-0.27	0.3
FMA (^o^)	-0.09	-0.25	0.1	-0.85	0.41	-0.31	0.13
OP-MP (^o^)	-0.04	-0.25	0.05	-0.95	0.36	-0.14	0.05
LI1-MP (^o^)	0.02	0.16	0.03	0.61	0.54	-0.05	0.08
L6-MP (mm)	0	-0.01	0.1	-0.05	0.96	-0.22	0.21
U6-pal (mm)	0.08	0.17	0.1	0.8	0.43	-0.13	0.29
WALA-M (mm)	-0.64	-0.49	0.28	-2.31	0.03*	-1.24	-0.05
OJ (mm)	-0.01	-0.02	0.08	-0.11	0.91	-0.17	0.15
OB (mm)	-0.04	-0.07	0.18	-0.24	0.81	-0.43	0.34
Number of observations	84						
ANOVA effect	df: 13; F: 1.74; p: 0.108						
Model summary	R (correlation coefficient): 0.79; R2 (R-squared): 0.62; adjusted R²: 26.18						

## Discussion

Andrews described six characteristics of normal occlusion and reported from his studies of 120 untreated subjects with pleasing appearances and correct bites that the COS ranged from a flat to a mild curve [3]. He concluded that the best intercuspation occurred when the OP was relatively flat. He also believed there was a natural tendency for the curve to deepen with time, causing the crowding of mandibular incisors. He proposed that flattening the OP should be a treatment goal as a form of overtreatment. The RCS wires are the most commonly used wires for leveling COS, and the mechanisms of the RCS in flattening the curve are less detailed in the existing literature. Further, Class II malocclusion showed an increasing depth of the Spee curve compared to other malocclusions [2,12]. The effect of leveling of COS and its correlation to different skeletal and dental parameters and dental-basal bone arch distance were assessed in the previous studies [13-15].

In the present study, we have tried to predict the relation between COS and 21 other parameters in non-growing individuals and patients with class II malocclusion to observe how the leveling of COS affects skeletal and dental factors and also how the parameters are correlated with the leveling of COS when non-extraction methods are used. Non-growing individuals are chosen to offset the effects of the growth changes. Further, we have chosen non-extraction criteria to assess the effects of RCS wires on the dentition in all three dimensions. We specifically selected the cephalometric landmarks in this study to assess potential correlations between the Spee curve and skeletal and dental parameters. This allowed us to explore the relationship between the flattening of the COS and its subsequent impact on the OP.

We observed higher COS values in average growers, which is, however, not statistically significant when compared to horizontal and vertical growers. The reduction in the depth of COS is proportional to the pre-treatment values, irrespective of growth patterns (Table 2). In our sample, males exhibited a relatively deeper COS (3.14 ± 0.79), however, without much variation from that of the females (2.89 ± 0.8). The present findings are in harmony with the previous studies, which demonstrated that this occlusal curvature lacks sexual dimorphism [14,16-18]. The crowding dentition exhibited a COS that was slightly greater than the spaced dentitions.

In the current study, the depth of the Spee curve (Table 3) showed a significant decrease in the mean COS from 2.97 mm pre-treatment to 1.54 mm post-treatment (p < 0.001*) after leveling. This is consistent with the findings of Fawaz et al. [15], who conducted a study to assess the depth of COS before and after treatment. The study revealed a mean COS of 3.13 mm before treatment and 0.83 mm post-treatment, indicating a highly significant difference in the depth of COS reduction in class II patients. According to Bernstein et al. [19], the mean changes observed in class II Div 1 cases treated by continuous mechanics were 2.28 mm, whereas another reported an average reduction of only 0.94 mm [9].

Skeletal parameters

We assessed whether there were any adaptive changes in the skeletal parameters during the leveling phase of COS (Table 3, Table 4). The sagittal skeletal parameters SNA (0), SNB (0), and ANB (0) exhibited adaptations that are of either no clinical significance or statistical significance. This is in quiet contrast to the previous study, which demonstrated a statistically significant decrease in ANB angle due to treatment by 2.98 ± 1.55. This is equivalent to a 57.75% decrease in the size of this angle [19]. We have found an inconsequential negative correlation between these parameters and the decrease in COS.

We observed a nugatory change in the increase of absolute values of FMA (0) by only 0.5 degrees with the alleviation of COS. A previous investigation of class II patients reported a mean increase of 3.52 degrees of FMA for a total mean reduction of COS of 2.3 mm [15]. They reported a negative correlation of -0.56 between FMA and COS. Their study did not mention the method of leveling the COS, but we utilized RCS wires. In our study, the positive direction of this parameter (0.22) with the reduction of COS was noticed.

OP alterations following the flattening of COS were noted in our study, which are clinically significant. The SN-OP (0) decreased (-3.05 ± 4.06), whereas the OP-MP (0) increased (1.82 ± 4.59) following the flattening of the COS. The findings support the study of Shannon and Nanda [9], who, in a mixed sample of different malocclusions, reported an increase in the OP-MP (0) by an average of 1.06 ± 2.95 with treatment. In contrast, the OP-SN angle decreased by 1.35° ± 2.76° following the reduction of COS. Similarly, another study on class II Div I cases exhibited a mean reduction of 2.98° ± 3.09° of SN-OP angle and an increase in OP-MP angle of 3.90 ± 3.83, and both changes were statistically significant (p < 0001) [19].

Relatively, the OP-MP (o) in the present study exhibited a stronger positive correlation (0.23 > 0.07) compared to that of the SN-OP (o), indicating a stronger association with COS, however, both without any statistical significance. There are controversial findings in the literature regarding the strength of the relationship between the SN-OP (o) and COS. The results of the present investigation align with a previous study that observed a tenuous association for SN-OP [9]. The study of Bernstein [19] could find a statistically significant correlation (P <.0001) between the two variables. However, both studies noted a significant positive correlation between the reduction of COS and the OP-MP (o) [9,19].

We also evaluated how the inclination of the CG affects the leveling of the COS. There is an insignificant increase in CG (0) from pre-treatment (43.74 ± 6.25) to post-treatment (45.18 ± 5.2). We observed a very weak positive correlation (0.05) between these two variables. However, CG is a fixed variable, and the findings should be interpreted carefully as they have functional significance. The usual range of this value is 22-60 degrees [10]. The two factors that determine the movement of the mandible in a forward direction are the incisal guidance angle (IGA) and the sagittal CG angle. The IGA is determined by the vertical overlap (overbite) and horizontal overlap (overjet) between the teeth [20]. In natural teeth, these dimensions of overbite and overjet are determined by the positions and the inclination of the teeth within the jaws. The treatment mechanics alter the incisal guidance during deep bite correction. The orthodontist should verify that these alterations in the incisal guidance are in harmony with the given CG of the patient. The study is the first to correlate CG and COS’s relationship. No study has been reported to date that measured CG before and after the leveling of COS and can be considered a parent reference for future studies.

Dental parameters

The study assessed the dental factors contributing to the correction of the COS (Table 3, Table 4). It included both the angular and linear parameters.

Angular measurements

A mean proclination of 2.48 degrees for lower incisors was noted, increasing the L1-MP (o) from 103.25 ± 9.1 of pre-treatment values to 105.73 ± 6.83 after treatment. There is also a decrease in the L6-MP (o) angle (-2.14 ± 5.64), suggesting distal inclination of the crowns of the first molar. This is similar to a previous study’s findings that demonstrated uprighting the first molar by 3.96° ± 5.71° following the reduction in the depth of COS [9]. We observed that LI-MP (o) is moderately correlated (-0.36) to the reduction of COS in a negative direction, with the p-values (0.06) close to those of statistical significance. This indicates an increase in the lower incisor inclination as the COS flattens. This finding is consistent with the previous studies [13,19]. Quiet contrastingly. AlQabandi et al. [21] found no significant association between the decrease in the depth of the Spee curve and the forward inclination of the lower incisor. Nevertheless, they discovered a strong correlation between the forward inclination of the lower incisors and a decrease in the ICW, as well as a decrease in dental crowding.

The L6-MP (o) decreased from pre-treatment values of 100.42 o to 98.28 o during the study period, indicating distal tipping or uprightning of molars. However, a weak negative correlation (0.07), which is statistically not significant, was found between L6-MP (o) and COS. These findings concord with the previous study suggesting that the mesial inclination of the molar tends to deepen the COS and vice versa [9].

Linear parameters

The linear parameters L1-MP (mm) and L6-MP (mm) give an insight into the absolute intrusion of incisors and the extrusion of molars. We observed extrusion of molars as measured by L6-MP by 0.78 ± 2.03 mm and an absolute intrusion of incisors (L1-MP) by -0.75 ± 2.23 mm.

The L1-MP (mm) has a relatively weak positive correlation (0.02) to the COS compared to the negative correlation (-0.27) of the L6-MP (mm). The L1-MP (mm) changes related to COS observed in the current study are consistent with Shannon and Nanda’s study [9]. In a mixed sample of malocclusion during leveling of COS, they reported an average extrusion of the mandibular first molar by 2.33 ± 1.58 mm, and the mandibular incisors were intruded at an average of 0.20 ± 2.28 mm with treatment and flared by 0.50° ± 6.93°. They also observed no significant changes but a positive correlation between lower incisor inclination and the COS. This indicates that molar extrusion has a more significant impact on COS reduction than absolute incisor intrusion, which is consistent with the findings of another study by Bernstein et al. [19]. However, they established a significant change in L1-MP (<0.0001) that contributes to the correction of COS [19].

PM-MP (mm) represents the alterations in the vertical displacement of the premolar. Typically, this corresponds to the COS measured on the dental casts as the deepest point from the premolars to the OP. The PM-MP treatment changes are statistically significant (0.001). The premolars exhibited greater extrusion than the molars, indicating the area where most of the curve correction occurred. We note a nonsignificant positive correlation (0.12) between this parameter and COS, which aligns with the results of the previous study that used the continuous archwire technique to treat class II Div 1 cases [19]. The study concluded that this technique is effective in non-extraction cases when the initial COS is 2-4 mm and leveling occurs by a combination of premolar extrusion and, to a lesser extent, incisor intrusion.

The current study also analyzed the change in the transverse dimensions of the mandibular arch, contributing to the reduction of COS. The ICW and the IMW displayed a statistically significant increase in the mean width at 2.5 mm and 2.93 mm, respectively. However, we noted a very weak positive correlation (0.01, 0.1) between these transverse dimensions and those of COS. The findings are discordant with the previous study, which demonstrated no significant change in inter-canine width after leveling of the COS [9]. No previous studies are available demonstrating the relationship between IMW and COS.

We also looked at the WALA-FA in terms of its transverse dimensions. WALA is the most noticeable spot in the keratinized gingiva, close to the mucogingival interface. The convergence of these points forms a prominent crest, which is clinically detectable and significant because it signifies the underlying bone structure and the anatomical boundary that governs tooth movement. According to Lundstrom AF [22], the facial axis point (FA point) is the most conspicuous point on the visible part of the tooth (clinical crown). The imaginary line that connects all the FA points determines the form of the dental arch. This study evaluates the link between the dental arch and basal arch, specifically known as WALA to FA or WALA-FA [23,24]. Andrews proposed that shaping archwires to this specific point would widen the dental arches by tilting teeth towards an upright posture centered inside the basal bone [24]. During the expansion of the arch, there are changes in the width of the arch that affect the Wilson curve. However, any changes in the dental arch’s width should remain within the width of the basal arch, or, in other words, within the alveolar housing.

Dindaroğlu et al. [1] conducted a prior study that found no significant relationship between the depth of the COS, the curve of Wison, and the WALA-FA distance in pretreatment mandibular models. In contrast, in the present study, WALA-FA distance in the premolar region (WALA-PM) increased by a mean value of 0.11 mm, whereas WALA-M decreased in the molar area by a mean value of -0.5 mm. Both the parameters showed a positive correlation with COS, and WALA-M showed a significant positive correlation (r = 0.4; p = 0.03). Our study is likely the first to quantify the distance between WALA and FA before and after COS leveling. The Wilson curve is a natural mediolateral curve that contacts the buccal and lingual cusp tips on each side of the arch due to the mandibular posterior teeth's natural lingual inclination. As a result, lingual cusps are lower than buccal cusps on the mandibular arch [20,25]. This alignment has two functional implications. One is that it provides resistance to loading; the second is that it provides masticatory efficiency. The increase in transverse dimensions caused by the RCS wires may vary depending on the basal arch width available, which may vary in different malocclusions, as reported in earlier studies [2,23]. Any alteration in the transverse dimension will alter the current inclination, causing the buccal cusps to flare out in the lower arch, leading to a decrease in Wilson’s curvature, an area that requires additional investigation.

In the present study, the overjet (OJ) and overbite (OB) also exhibited a reduction of -1.67 and -1.51 mm, respectively. In the present study, overjet (r = 0.39; p = 0.038) and overbite (r = 0.57; p = 0.02) showed a significant positive correlation with the change in the depth of COS. The findings corroborate a previous study's conclusion that the curve’s variation significantly influences the amount of overjet and overbite [26].

A multiple linear regression model was analyzed by entering all the categorical values and predetermined continuous variables with coefficient values above 0.2 and those that exhibited statistical significance to predict the changes in the dependent variable, the COS. The following regression model was predicted: COS = {2.75 + (0.1 * Alignment- spacing) + (- 0.04 * FMA-nominal-Average) + (- 0.66 * FMA-nominal - Vertical) + ( + 0.16 · Gender- Male) +(+ 0.02 * SNB) + (- 0.09 * FMA (o) + (- 0.04 * OP-MP (o) + (+ 0.02 * LI-MP (o) + (-0 * L6-MP (mm) + (+0.08 * U6-pal(mm) + (-0.64 * WALA-M) + (-0.01 * OJ (mm) + (-0.04 * OB (mm)}.

The regression output for the average and vertical growth patterns shows a negative value, suggesting that these two groups have a lower inclination to influence COS decrease than the average growth patterns. In contrast, crowded dentitions are more likely to decrease the COS than spaced dentitions. Nevertheless, this prediction lacks statistical significance. The standardized coefficient beta is unaffected by the measured variable and always falls within the range of -1 to 1. The magnitude of the beta coefficient positively correlates with the extent to which the independent variable contributes to explaining the dependent variable, COS. The variable “WALA-M” exerts the most significant impact on the variable COS, as seen by its standardized coefficient beta value of -0.49. The p-value for the coefficient of the variable WALA-M was 0.03. The model’s R (correlation coefficient) value is 0.79, indicating a strong positive connection between the observed values and the model’s predictions. The model summary suggests a decent fit, with an R-squared value of 0.62, indicating that the predictors included in the model can account for about 61.72% of the changes in the COS. The adjusted R², which considers the number of variables in the model and the number of observations, indicates that approximately 25.45% of the variation in the dependent variable can be explained after adjusting for the number of predictors.

Extraction and non-extraction treatments may produce different effects on the curve. The non-extraction mode in our study resulted in the flaring of mandibular incisors and an increase in the ICW, similar to the previous study [9]. Only one comparison study investigated the impact of the RCS archwires versus anterior bite turbos in the treatment of deep overbite [27]. Their sample included mild class II and class I cases with a deep bite and sufficient overjet to place turbos on the lingual inclines of maxillary anterior teeth. The study concluded that lower incisors exhibited greater proclination and significant distal tipping when treated with RCS wires compared to those treated with bite turbos. Specifically, a 1 mm absolute intrusion of the lower incisors was observed in cases treated with 0.016 × 0.022 NiTi RCA, whereas only 0.28 mm of intrusion was noted in cases treated with bite turbos.

Most published studies reported alterations in the depth of the COS during the treatment, with only intra-arch anteroposterior changes. This is the first study to investigate the correlation between the COS and all the variables in all three dimensions that orthodontic procedures can modify. Many different treatment modalities are available for leveling the curve and opening the bite. Many of the previous studies did not attempt to qualify the treatment methods, whereas our study included the RCS archwires. An in-depth analysis was conducted to examine the impact of therapy on various areas of the arch.

The study’s results may give an insight into the type of deep bite cases that need to be selected for leveling the COS with NiTi RCS wires. The cases with already proclined lower incisors and distally tipped lower molars should be carefully selected, as this may increase the lower facial height. Further, the basal arch width available for a given transverse dimension of the arch should be carefully assessed before putting up the RCS wires.

Limitations of the study

The study was conducted only on class II malocclusions with specifically defined skeletal and dental characteristics. It is not appropriate to extrapolate the results of this study to the other malocclusion groups. Therefore, further studies are needed to investigate the correlation between the depth of the reduction of COS and other parameters in different malocclusion groups. The categorical analysis of the effect of the COS was done post-sample allocation. The study’s limited number of predictor observations led to a low variance in the adjusted R2 values of the regression model. This suggests a large sample size with appropriate categorical allocation to validate the model. This study included only the age groups not in the active growing phase. The issue of directionality arises when two variables exhibit correlation and potentially have a causal connection. Still, it is not feasible to determine which variable is responsible for inducing changes in another. Furthermore, because there are moderating variables, the “predictors of” and “determinants of” COS cannot be clearly defined.

## Conclusions

Within the constraints of the study, it can be concluded that effective leveling of the COS by RCS wires is achieved by a combined proclination of the lower anteriors and distal tipping of the molars. The linear dental changes included the vertical eruption of premolars and molars and the intrusion of lower anteriors. The increase in transverse arch widths contributes to the correction of deep bites. The flattening of the COS shows a notable impact on the orientation of the OP.
